# Supplementary material for: Accuracy and Clinical Impact of Estimating Low-Density Lipoprotein-Cholesterol at High and Low Levels by Different Equations
Source: Biomedicines. 2022 Dec 6;10(12):3156. doi: 10.3390/biomedicines10123156 (PMC9776049; doi:10.3390/biomedicines10123156)
Supplement: Supplementary file 1 [file biomedicines-10-03156-s001.zip › biomedicines-2050930-supplementary.pdf]

Supplemental Table S1. **Lipid values and demographic characteristics of dataset**

	min	max	Median (IQR)	Mean (SD)
ApoB*	5	377	96 (78-117)	99.4 (31.9)
HDL-C	2	201	44 (36-54)	46.2 (15.3)
TC	27	819	191 (159-227)	196.4 (56.2)
TG	5	1984	131 (89-207)	181.2 (172)
BQ-LDL-C	7	678	113 (88-142)	117.8 (44)
NonHDL-C	12	765	143 (113-179)	150.3 (54.8)
F-LDL-C	-308.2	721.8	110 (83-141)	114 (48.8)
M-LDL-C	-84.3	724.2	115 (90-145)	120.1 (46)
eM-LDL-C	-45.8	724.2	115 (90-145)	120 (45.8)
S-LDL-C	-14.1	699.6	113 (87-144)	118.2 (45.7)
AGE	18	90	55 (45-65)	54.6 (15)

% Male = 51.5   n = 39,956 *n=24,463

Supplemental Table S2. **Equations for calculating LDL-C**

$$Friedewald\ LDLC = TC - HDLC - \left(\frac{TG}{5}\right)$$

$$Martin\ LDLC = TC - HDLC - \left(\frac{TG}{factor}\right)$$

$$Sampson\ LDLC = \frac{TC}{0.948} - \frac{HDLC}{0.971} - \left(\frac{TG}{8.56} + \frac{(TGxNonHDLC)}{2140} - \frac{TG^2}{16100}\right) - 9.44$$

Supplemental Table S3. Comparison between equations for accuracy

TG 0-400 mg/dL					
**Response1**	**Response2**	**Kappa**	**Std Err**	**Bowker Symmetry ChiSquare**	**Bowker P-Value**
**BQ LDL-C <70 mg/dL**					
S LDL -C	F LDL -C	0.9098	0.0031	653.9	<0.0001
S LDL -C	M LDL -C	0.9358	0.0027	240.1	<0.0001
F LDL -C	M LDL -C	0.8504	0.0039	885.0	<0.0001
**BQ LDL-C <190 mg/dL**					
S LDL -C	F LDL -C	0.9668	0.0027	146.0	<0.0001
S LDL -C	M LDL -C	0.9379	0.0036	39.6	<0.0001
F LDL -C	M LDL -C	0.9565	0.0031	34.6	<0.0001
**TG 401-800 mg/dL**					
**Response1**	**Response2**	**Kappa**	**Std Err**	**Bowker Symmetry ChiSquare**	**Bowker P-Value**
**BQ LDL-C <70 mg/dL**					
S LDL -C	F LDL -C	0.6731	0.0145	359.0	<0.0001
S LDL -C	M LDL -C	0.6896	0.0146	265.0	<0.0001
F LDL -C	M LDL -C	0.4243	0.0136	624.0	<0.0001
S LDL -C	eM LDL -C	0.6768	0.0146	276.0	<0.0001
F LDL -C	eM LDL -C	0.4147	0.0134	635.0	<0.0001
M LDL -C	eM LDL -C	0.9415	0.0083	2.7	0.8454*
**BQ LDL-C <190 mg/dL**					
S LDL -C	F LDL -C	0.9544	0.0112	16.0	0.0138
S LDL -C	M LDL -C	0.6943	0.0232	133.0	<0.0001
F LDL -C	M LDL -C	0.7368	0.0219	117.0	<0.0001
S LDL -C	eM LDL -C	0.7290	0.0226	113.0	<0.0001
F LDL -C	eM LDL -C	0.7727	0.0210	97.0	<0.0001
M LDL -C	eM LDL -C	0.9563	0.0093	18.2	0.0058

Supplemental Figure S1

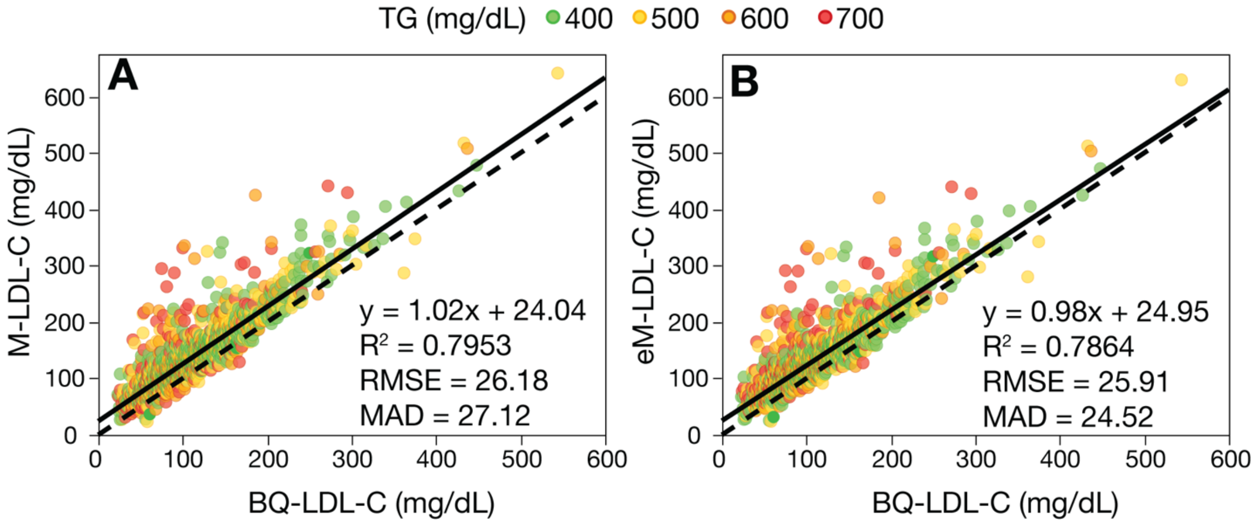

Figure S1. Comparison of estimated LDL-C versus BQ-LDL-C. LDL-C was calculated for patients (N=2267) with a wide range of LDL-C values and TG 400–800 mg/dL by the M-LDL-C (panel A), and eM-LDL-C (Panel B) and plotted against LDL-C as measured by BQ reference method (BQ-LDL-C). Solid lines are the linear fit for indicated regression equations. Dotted lines are lines of identity. Results are color coded by TG level with the value in the legend (mg/dL) indicating the start of each interval.

Supplemental Figure S2

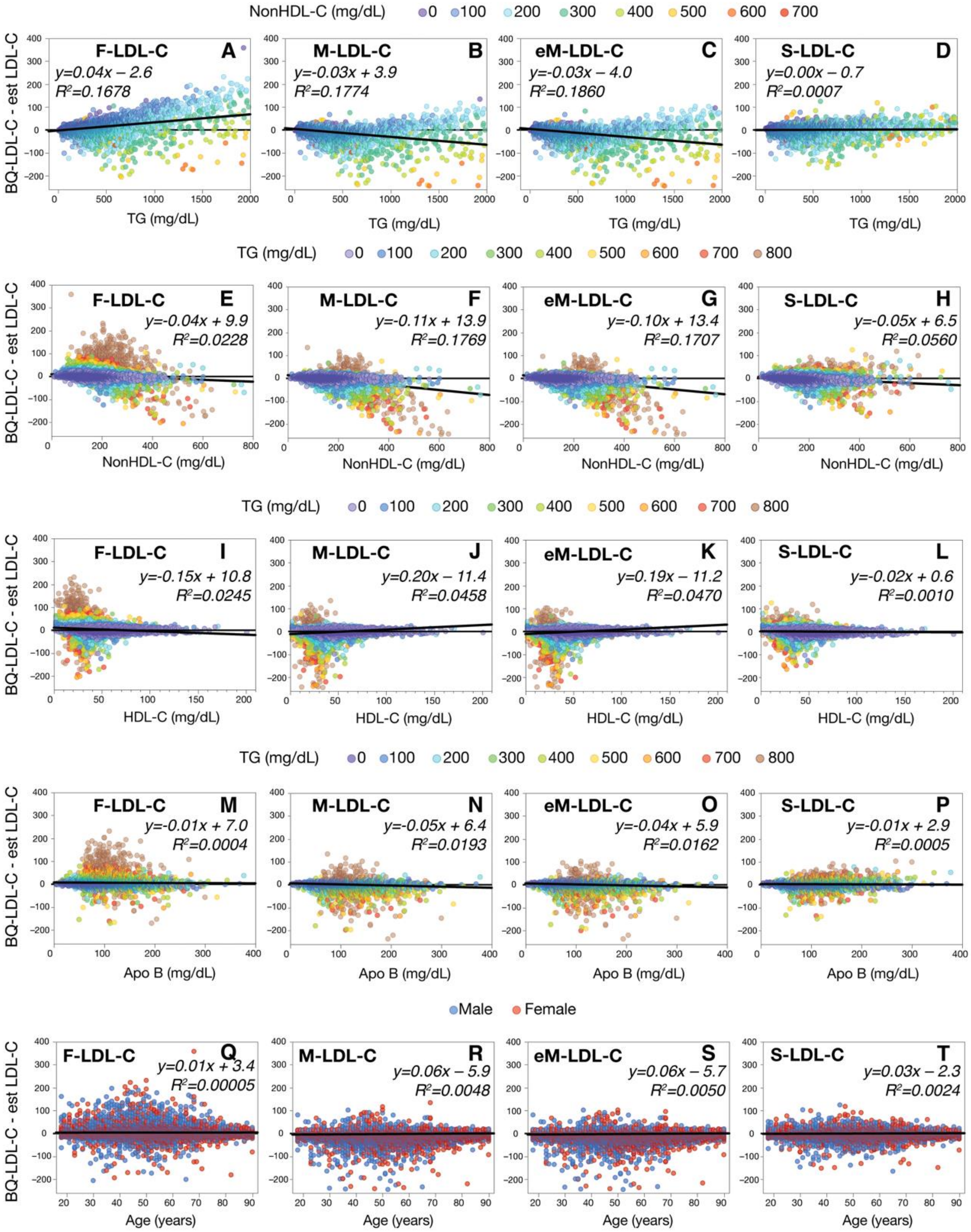

Figure S2. Residual error plots of estimated LDL-C versus BQ-LDL-C. LDL-C was calculated from the results of a standard lipid panel from a general population (N=39,956) with a wide range

of LDL-C values by F-LDL-C (Panel A, E, I, M and Q), and M-LDL-C (Panels B, F, J, N and R), eM-LDL-C (Panels C, G, K, O and S) and S-LDL-C (Panels D, H, L, P and T). The difference from LDL-C as measured by the BQ reference method was plotted for the indicated independent variables. Results are color coded by nonHDL-C level (Panels A-D), triglyceride level (Panels E-P) with the value in the legend (mg/dL) indicating the start of each interval or by sex (Panels Q-T, blue = male, red = female).

Supplemental Figure S3

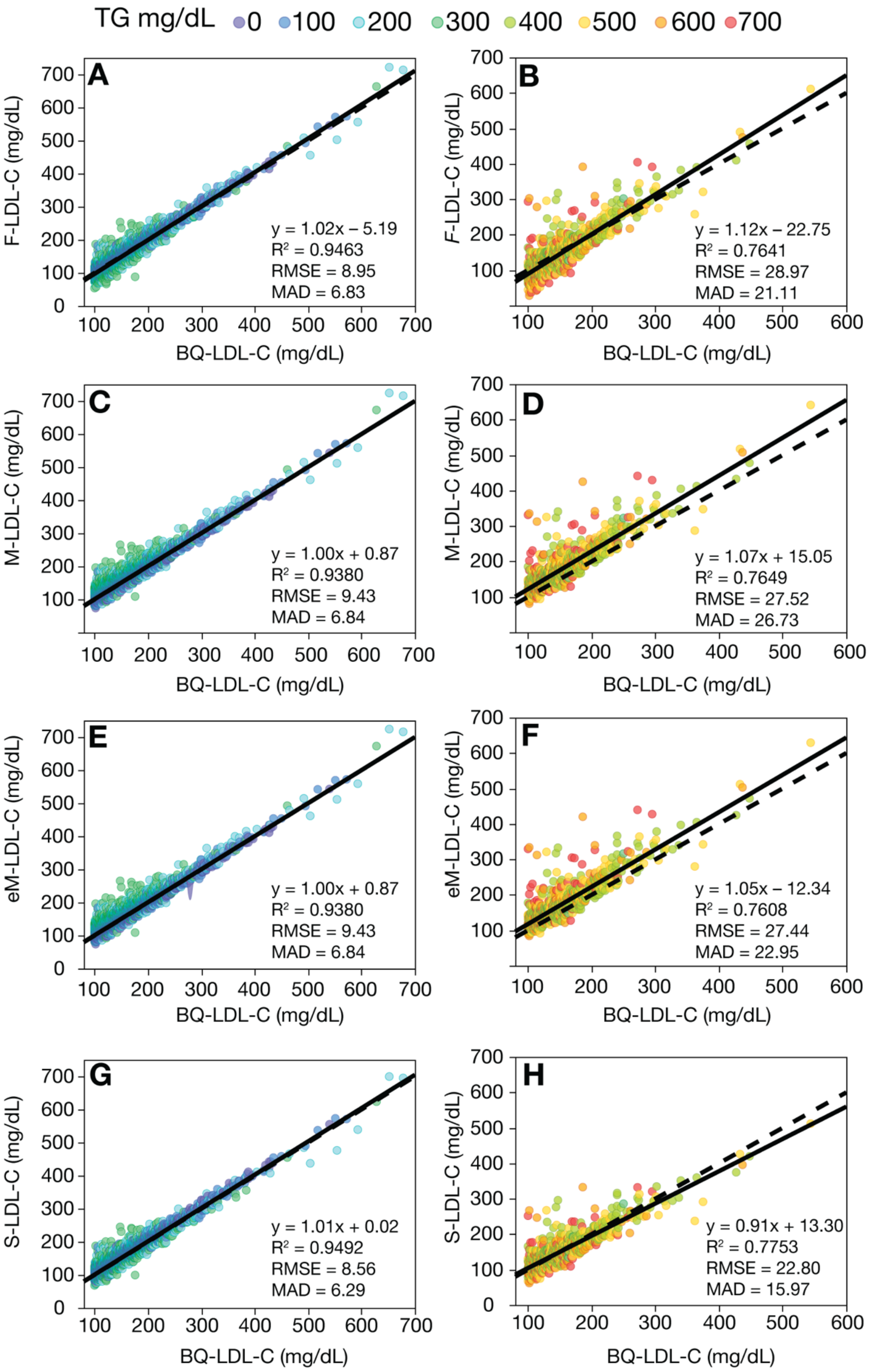

Figure S3. Comparison of estimated LDL-C versus BQ-LDL-C. LDL-C was calculated from the results of a standard lipid panel from a general population (N=25,311) with a wide range of LDL-C >100 mg/dL by F-LDL-C (Panels A, B), M-LDL-C (Panels C, D), eM-LDL-C (Panels E, F) and *S*-LDL-C (Panels G, H) equations and plotted against LDL-C as measured by BQ reference method (BQ-LDL-C) for low TG <400 mg/dL (N=24,142,Panels A, C, E G) and for high TG 400–800 mg/dL (N=1169, Panels B, D, F and H). Solid lines are the linear fits for theindicated regression equations. Dotted lines are lines of identity. Results are color coded by TG level with the value in the legend (mg/dL) indicating the start of each interval.
